# Metal Based Drugs Restyled and Resumed

**DOI:** 10.1155/2007/16260

**Published:** 2008-03-25

**Authors:** Gianni Sava

**Affiliations:** Department of Life Sciences, University of Trieste, via L. Giorgieri 7, 34127 Trieste, Italy

This is the first issue of Metal Based Drugs, our journal, restyled and resumed after 5 years' silence. A number of distinguished scientists have agreed to be Associate Editors and to assist in the new course of this multidisciplinary journal, to encourage it to survive and to be the forum where to post the most important findings of our intriguing research of new and innovative metal-based drugs. With your help, the journal has an excellent potential to rapidly become the most important reference in the biomedical literature of drugs for diseases such as cancer and diabetes or for diagnostics based on the modern molecular approach formetals in biology and medicine.

Undoubtedly, of the many fields where metals can help medicine with innovative drugs, chemotherapy and particularly cancer chemotherapy is forever attracting considerable interest. In this context it is important to note that there is a decreasing interest in DNA-damaging drugs. Unlike the era initiated with the discovery of the anticancer activity of mechlorethamine in 1940s, it is more commonly accepted that the new golden era of cancer chemotherapy is driven by a new series of drugs characterized by their capacity to interact with targets expressed with high selectivity only by tumour cells and this is now giving rise to drugs that are actually available in clinical practice. It is clear that the “old drugs” will still accompany us in the clinical treatment of tumours for many years. It is similarly evident that the few improvements we may expect from these drugs (those acting on nucleic acids or on macromolecules of the mitotic spindle) are those associated with a better pharmacokinetic profile that can lead to the reduction of systemic toxicity, the main problem limiting the use of such drugs.

Researchers are now convinced that targets such as DNA, RNA, and tubulin do not provide sufficient selectivity for cancer cells without negative effects on healthy tissues. Also, factors such as the high tendency to develop resistance towards chemotherapeutic agents, the genetic instability of tumour cells, their heterogeneity, and their elevated mutation index are elements sufficient to circumvent the practical application of chemotherapy. Furthermore, we now have new ways to develop and research ideas on the processes of gene regulation involved in tumour malignancy: activation and repression of signal transduction pathways depending on various extracellular signals and/or cellmutations that select their capacity to survive, generate tumours, and to invade and give rise to metastases, the ultimate target of chemotherapy.

We therefore have important work to do: to obtain new metal-based drugs that are not related to cisplatin or platinum analogues currently in use or in the advanced stages of development, since these represent part of the old strategy of antitumour chemotherapy. We are conscious of the difficulties of the game but we will run this race with the important advantage of having 40 years of accumulated experience with a number of metals and ligands, significant knowledge of the chemical behavior of these compounds in solution and in physiological media, and on their interactions with biological molecules. The open-access journal Metal Based Drugs represents an important aid to develop these topics and others, for example, on diabetes and on molecular diagnostics that we have not raised here but are equally important and represent the opposite side of the same coin.

*Good luck fellow colleagues* *Good luck fellow colleagues*

*Gianni Sava* *Gianni Sava*

